# Influence of compensation status on time off work after carpal tunnel release and rotator cuff surgery: a meta-analysis

**DOI:** 10.1186/1754-9493-7-1

**Published:** 2013-01-02

**Authors:** Vinícius Ynoe de Moraes, Katelyn Godin, João Baptista Gomes dos Santos, Flávio Faloppa, Mohit Bhandari, João Carlos Belloti

**Affiliations:** 1 Division of Hand and Upper Limb Surgery, Department of Orthopaedics and Trauma, Universidade Federal de São Paulo (UNIFESP-EPM), Rua Borges Lagoa, 778, São Paulo, Brazil; 2 Division of Orthopaedic Surgery, Department of Surgery, McMaster University, 293 Wellignton St. N, Hamilton, ON, L8L 8E7, Canada

**Keywords:** Workers’ compensation, Hand surgery, Outcomes, Carpal tunnel syndrome, Rotator cuff tears, Systematic review, Time to return to work

## Abstract

**Background:**

The assessment of post-surgical outcomes among patients with Workers’ Compensation is challenging as their results are typically worse compared to those who do not receive this compensation. These patients’ time to return to work is a relevant outcome measure as it illustrates the economic and social implications of this phenomenon. In this meta-analysis we aimed to assess the influence of this factor, comparing compensated and non-compensated patients.

**Findings:**

Two authors independently searched *MEDLINE (Ovid), Embase (Ovid), CINAHL, Google Scholar, LILACS and the Cochrane Library* and also searched for references from the retrieved studies. We aimed to find prospective studies that compared carpal tunnel release and elective rotator cuff surgery outcomes for Workers’ Compensation patients versus their non-compensated counterparts. We assessed the studies’ quality using the Guyatt & Busse Risk of Bias Tool. Data collection was performed to depict included studies characteristics and meta-analysis. Three studies were included in the review. Two of these studies assessed the outcomes following carpal tunnel release while the other focused on rotator cuff repair. The results demonstrated that time to return to work was longer for patients that were compensated and that there was a strong association between this outcome and compensation status - Standard Mean Difference, 1.35 (IC 95%; 0.91-1.80, p < 0.001).

**Conclusions:**

This study demonstrated that compensated patients have a longer return to work time following carpal tunnel release and elective rotator cuff surgery, compared to patients who did not receive compensation. Surgeons and health providers should be mindful of this phenomenon when evaluating the prognosis of a surgery for a patient receiving compensation for their condition.

**Type of study/level of evidence:**

Meta-analysis of prospective Studies/ Level III

## Introduction

Numerous studies have demonstrated that Workers’ Compensation patients have a longer return to work time compared to non-compensated patients [1-3]. Other studies have described the inherent difficulties associated with measuring time to return to work as an outcome of the success of a surgical treatment [4-6].

These findings are of relevance when a surgeon is attempting to predict a patient’s loss of working days and the accompanying economical and social implication [7]. There are numerous factors that have been shown to determine return to work time including psychological issues, job type and work-place features [1,4,5,8,9].

We believe that is important to understand and quantify the difference in return to work time between compensated and non-compensated patients. We did not recognize this approach based in a Systematic Review and Meta-Analysis including only a best-evidence approach.

This systematic review and meta-analysis aims to analyze time to return to work as reported from data gathered from prospective studies that assessed patient outcomes following carpal tunnel release and elective rotator cuff surgery.

## Methods

This study is a complementary analysis from a broader systematic review that assessed the results of the compensation status following orthopedic surgery [10] and considered functional outcomes as the main endpoint. The endpoints assessed in this report were set a priori and the protocol was published before study began in a prospective database for systematic reviews (http://www.crd.york.ac.uk/prospero) under the record number CRD42012002121 [11].

### Search strategy and assessment of eligible studies

The following databases were searched: *MEDLINE (Ovid), Embase (Ovid), CINAHL, Google Scholar, LILACS and the Cochrane Library*. We also hand-searched the references sections of these primary papers in order to locate additional studies and to avoid missing relevant papers. We did not exclude any studies on the basis of language.

**Figure 1:**
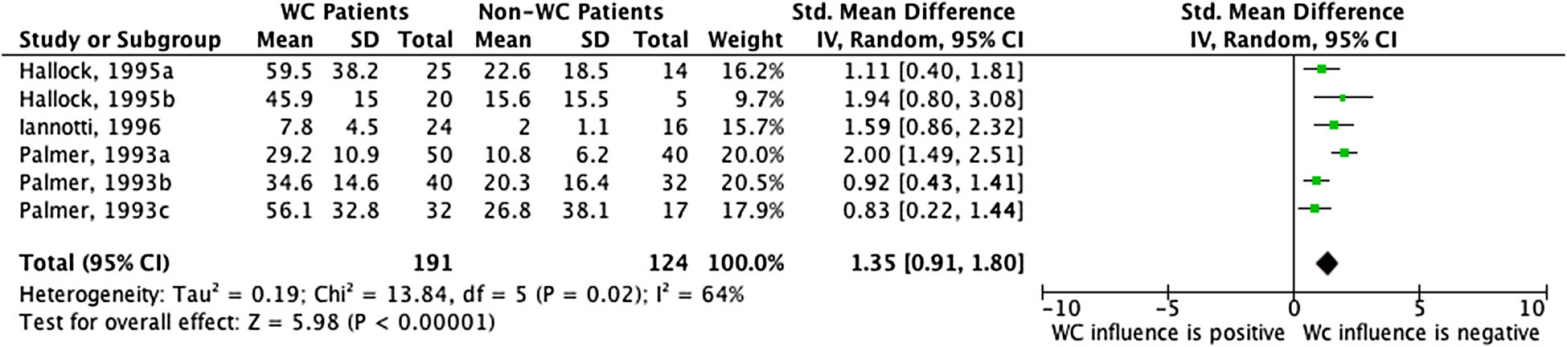
**Forest Plot – Continuous data comparison between compensated and non-compensated patients.** WC = Worker’s compensation. Letters a,b,c refers to different cohorts/interventions for the same study.

We included papers published between 1992 and 2012 (May, 2012). Our search strategy is shown in Figure 1.

### Selection criteria

Studies were included if they met the following criteria: 1) the data was collected and analyzed prospectively; 2) the authors assessed the influence of compensation status specifically; 3) orthopaedic surgery was the main intervention. In this report, we included only the studies that reported time to return to work after upper limb orthopaedic surgery (carpal tunnel release and elective rotator cuff surgery). We excluded studies that: 1) involved non-surgical treatments; 2) studies that collected patient data retrospectively; and 3) the study did not report any of the outcomes of interest, as described above. We included studies after a 2-stage assessment. Disagreements regarding which studies should be included were resolved by group discussion (VY and KG).

### Data management: collection and extraction

Quantitative data was extracted following full text analysis of the included studies. Other important information that was extracted included details on the study design, funding, intervention, control (if applicable) and outcomes.

We collected data (VY, KG) as continuous and analyses were performed in forest plots. Time to return to work data was collected from reported means, standard deviations, and the number of patients in each group. In the event that the published data was missing and/or unclear, we also attempted to contact the authors by email to clarify or provide us with additional data from their study. Following data entry, all data was verified by two authors (VY and JB).

### Study quality assessment

We assessed the quality of all included studies using a specific tool [12] developed to appraise the risk of bias within observational studies. Each included study was graded on a four-category scale according to their risk of bias. The scale and its grading scheme is shown in Additional file 1: Appendix 1.

### Statistical analysis

We utilized RevMan [13] (version 5.1) to conduct the meta-analysis. The data was summarized using an Inverse Variance method in a Random Effect model. We provided measures as standardized mean difference [14], as proposed by Hedges. We followed Cohen’s interpretation on the magnitude of effect size: small effect < 0.20, medium effect < 0.50 and large effect < 0.80. We provided 95% confidence intervals shown in forest plots for each of the included studies and also for the sum of the studies [14]. Heterogeneity was assessed by I^2^ statistics and is depicted in a forest plot.

## Results

**Figure 2:**
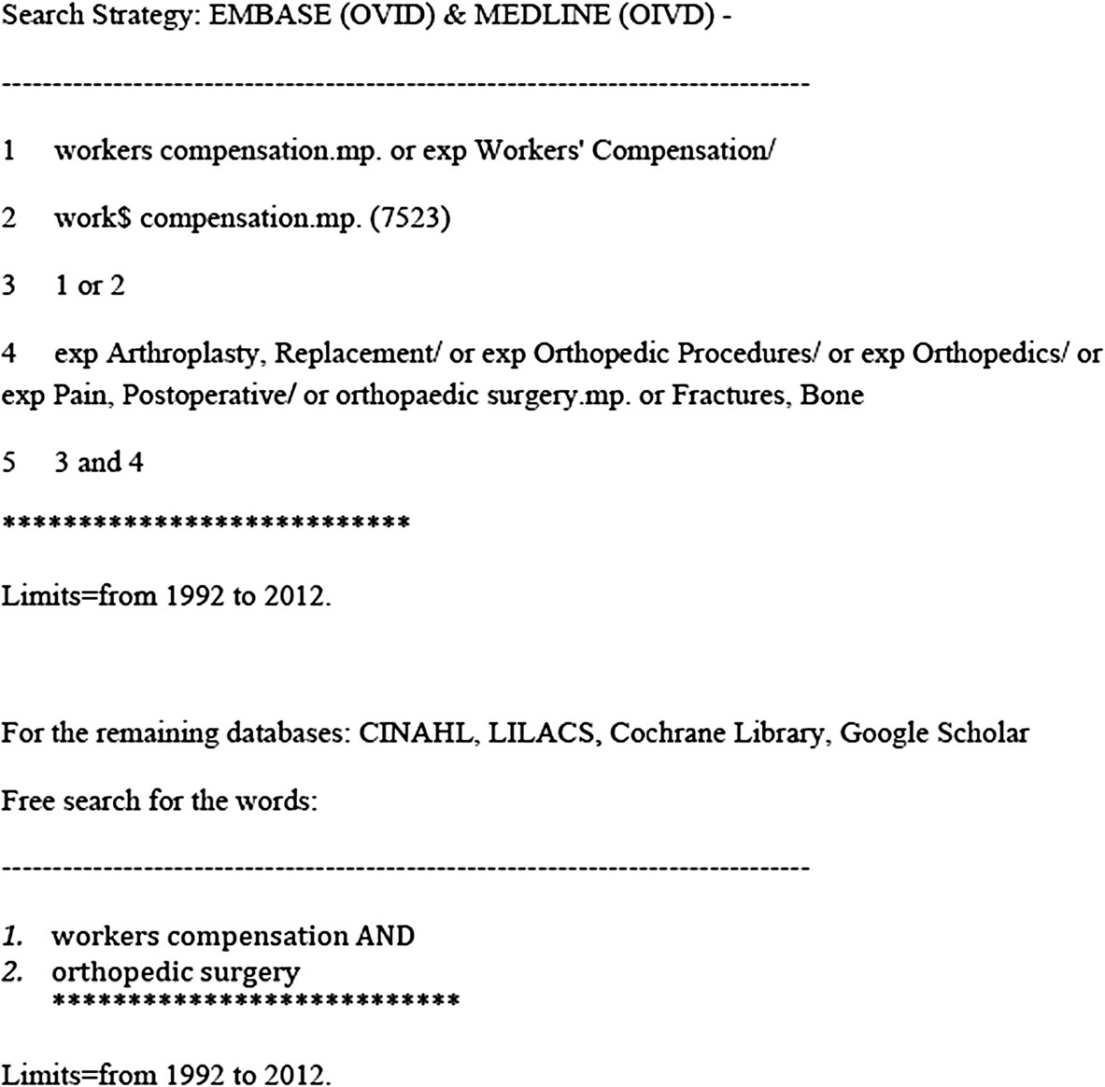
Search strategy.

Using our search strategy, we analyzed the title and abstract of 791 studies for relevance to our study. We assessed the full text of 67 studies and 20 met our inclusion criteria. We identified three studies that assessed time to return to work in patients who were compensated versus those who did not receive compensation. The analyses included 315 patients. The studies’ characteristics are presented in Tables 1 and 2. Two studies were excluded because they did not report the data we required. Despite our efforts to retrieve this information from authors, we could not obtain this data. The quality assessment demonstrated that methodological quality differed among studies. One study was evaluated as having a low risk of bias [3], one has as intermediate risk [15] and one as high risk [2]. Figure 2 demonstrates a strong effect size for the relationship between time to return to work and compensation status, with compensated patients having a longer return to work time.

**Table 1:** Studies characteristics: qualitative data

**Study**	**Intervention**	**Country**	**Study design**	**Funding**	
Palmer [15] 1993	Carpal Tunnel Release	US	Prospective Case Series	No	
Hallock [2] 1995	Carpal Tunnel Release	US	Prospective Case Series	No	
Iannotti [3] 1996	Rotator Cuff Tear Repair	US	Prospective Case Series	No	

**Table 2:** Studies characteristics: quantitative data

**Study**	**Participants (n)**	**Intervention**	**Gender (%)**	**Age (SD)**	**Follow up losses (%)**
Palmer [15]1993	163	Carpal Tunnel Release	73(44.7%)	42.2-46.8	0
Hallock [2] 1995	96	Carpal Tunnel Release	26(27%)	45.6(17)/ 42.6(14)	0
Iannotti [3] 1996	46	Rotator Cuff Tear Repair	31(77.5%)	55(11)	13

## Discussion

Our results demonstrate that compensated patients had a longer time of absence from work following orthopedic surgery. Although this finding may be intuitive, this study demonstrates this relationship using a rigorous, systematic perspective.

Some studies have previously demonstrated that time to return to work is not an optimal measure for surgeons when assessing post-surgery outcomes [4,16,17]. However, return to work time is an important consideration as it contributes to social and economical burden [6].

Factors that influence earlier or later return to work are well-established in the literature and typically relate to the particular health condition, surgical intervention, the surgeon’s approach for post-operative treatment and care and patient characteristics [1,4]. This study demonstrates that the presence of Worker’s Compensation is an additional factor that predicts a longer return to work period post-surgery.

This meta-analysis provides quantitative data as Standardized Mean Difference. This is not a directly usable measure, as it measures the standardized difference between the standard deviations between the groups of interest. This measure is frequently used in Cochrane Reviews when different scales are pooled in the same forest plot. In our study, this quantification allows us to state that compensated patients take longer to return to work compared to non-compensated patients and the magnitude of this effect is strong [14].

The small number of included studies and the lack of studies including different conditions reduced the external validity of our results as our findings may not be generalizable to other patient populations. We opted not to include lower limb diseases or acute injuries (e.g. fractures) since this would increase the heterogeneity of our results in an inacceptable manner for our outcome of interest. This study is strengthened by the robustness of the methodology, the strict inclusion criteria and quality assessment.

## Conclusions

Compensated patients are more likely to have longer periods of recovery after carpal tunnel release and elective rotator cuff surgery. This finding has significant clinical and economical implications. Prospective studies are the best way to determine which factors influence return to work time following surgery.

## Competing interest

No additional external funding received for this study. The authors declare that they have no competing interests. The funders had no role in study design, data collection and analysis, decision to publish, or preparation of the manuscript.

## Authors’ contributions

VYM and KG wrote and JCB reviewed the manuscript. VYM developed the idea for the study. VYM and KG carried out the study’s assessment. VYM performed the statistical analysis and MB provided research support. JBGS and FF provided additional research support and were involved in the manuscript proofreading. VYM and KG revised the manuscript. All authors have read and approved the final manuscript.

## Supplementary Material

Additional file 1 **Appendix 1.** Tool to Assess Risk of Bias in Cohort Studies. Click here for file

## References

[B1] Adams ML Franklin GM Barnhart S Outcome of carpal-tunnel surgery in Washington-state workers compensation Am J Ind Med 1994 25 527 536 10.1002/ajim.4700250407 8010295

[B2] Hallock GG Lutz DA Prospective comparison of minimal incision “open” and two-portal endoscopic carpal tunnel release Plast Reconstr Surg 1995 96 941 947 10.1097/00006534-199509001-00027 7652069

[B3] Iannotti JP Bernot MP Kuhlman JR Kelley MJ Williams GR Postoperative assessment of shoulder function: a prospective study of full-thickness rotator cuff tears J Shoulder Elbow Surg 1996 5 449 457 10.1016/S1058-2746(96)80017-6 8981270

[B4] Cowan J Makanji H Mudgal C Jupiter J Ring D Determinants of return to work after carpal tunnel release J Hand Surg-Am 2012 37A 18 27 22137062 10.1016/j.jhsa.2011.10.033

[B5] Parot-Schinkel E Roquelaure Y Ha C Leclerc A Chastang JF Raimbeau G Chaise F Descatha A Factors affecting return to work after carpal tunnel syndrome surgery in a large french cohort Arch Phys Med Rehab 2011 92 1863 1869 10.1016/j.apmr.2011.06.001 22032220

[B6] MacDermid JC Grewal R MacIntyre NJ Using an evidence-based approach to measure outcomes in clinical practice Hand Clin 2009 25 97 101 10.1016/j.hcl.2008.11.001 19232920

[B7] Mallick A Clarke M Wilson S Newey ML Reducing the economic impact of carpal tunnel surgery J Hand Surg-Eur Vol 2009 34E 679 681 19587079 10.1177/1753193409105578

[B8] Acharya AD Auchincloss JM Return to functional hand use and work following open carpal tunnel surgery J Hand Surg-Brit Eur 2005 30B 607 610 10.1016/j.jhsb.2005.06.018 16115707

[B9] Bruyns CNP Jaquet JB Schreuders TAR Kalmijn S Kuypers PDL Hovius SER Predictors for return to work in patients with median and ulnar nerve injuries J Hand Surg-Am 2003 28A 28 34 12563634 10.1053/jhsu.2003.50026

[B10] de Moraes VY Godin K Tamaoki MJS Faloppa F Bhandari M Belloti JC Workers’ Compensation status: does It affect orthopaedic surgery outcomes? A meta-analysis PLoS One 2012 7 e50251 10.1371/journal.pone.0050251 23227160 PMC3515555

[B11] Moraes VY Belloti JC Godin K Bhandari M Influence from presumed compensation status in orthopedic surgery: does it affect the outcomes? a systematic review of prospective studies PROSPERO Database 2012 CRD42012002121

[B12] Guyatt G Busse J Tool to assess risk of bias in cohort studies 2011 Hamilton, ON: In Book Tool to Assess Risk of Bias in Cohort Studies

[B13] RevMan R The nordic cochrane centre, the cochrane collaboration Book [computer program]. version 5.1. Copenhagen: The nordic cochrane centre 2011 Version 51 Copenhagen: The Cochrane Collaboration

[B14] Durlak JA How to select, calculate, and interpret effect sizes J Ped Psych 2009 34 917 928 10.1093/jpepsy/jsp004 19223279

[B15] Palmer DH Paulson JC Lanelarsen CL Peulen VK Olson JD Endoscopic carpal-tunnel release - a comparison of 2 techniques with open release Arthroscopy 1993 9 498 508 10.1016/S0749-8063(05)80396-2 8280321

[B16] Atroshi I Johnsson R Ornstein E Patient satisfaction and return to work after endoscopic carpal tunnel surgery J Hand Surg-Am 1998 23A 58 65 9523956 10.1016/S0363-5023(98)80090-7

[B17] Baldwin ML Johnson WG Butler RJ The error of using returns-to-work to measure the outcomes of health care Am J Ind Med 1996 29 632 641 10.1002/(SICI)1097-0274(199606)29:6<632::AID-AJIM7>3.0.CO;2-L 8773723

